# A retrospective study of deltoid ligament repair versus syndesmotic fixation in lateral malleolus fracture combined with both deltoid ligament injury and inferior tibiofibular syndesmotic disruption

**DOI:** 10.3389/fsurg.2022.912024

**Published:** 2022-10-20

**Authors:** Junyi Liao, Jinsong Zhang, Weidong Ni, Gang Luo

**Affiliations:** Department of Orthopaedic Surgery, The First Affiliated Hospital of Chongqing Medical University, Chongqing, China

**Keywords:** ankle fracture, deltoid ligament injury, inferior tibiofibular syndesmotic disruption, deltoid ligament repair, trans-syndesmotic fixation

## Abstract

**Background:**

To compare clinical outcomes of deltoid ligament repair versus syndesmotic fixation in lateral malleolus fracture combined with both deltoid ligament injury and inferior tibiofibular syndesmotic disruption.

**Methods:**

Patients diagnosed with lateral malleolus fracture combined with both deltoid ligament injury and inferior tibiofibular syndesmotic disruption who received open reduction and internal fixation (ORIF) were retrospectively reviewed. Seventy-eight patients were enrolled into the study, including 40 patients treated with lateral malleolus fracture ORIF and trans-syndesmotic fixation, and 38 patients treated with lateral malleolus fracture ORIF and deltoid ligament repair. Basic information and pre- and postoperative radiological materials were reviewed. Visual analog pain scale (VAS) score, Olerud–Molander score, and the American Orthopaedic Foot and Ankle Society (AOFAS) Ankle-Hindfoot Scale were used for evaluating pain control and functional recovery postoperatively at different time points.

**Results:**

No complication was reported in both groups. In the trans-syndesmotic fixation group, all patients received syndesmotic screw removal 6–8 weeks postoperatively. The Olerud–Molander score and AOFAS Ankle-Hindfoot Scale in the deltoid ligament repair group were higher than the trans-syndesmotic fixation group 3 months after operation. No statistical difference was found between the two groups in VAS score from 1 to 12 months postoperatively.

**Conclusions:**

Lateral malleolus fracture ORIF and deltoid ligament repair is an effective method for lateral malleolus fracture combined with both deltoid ligament injury and inferior tibiofibular syndesmotic disruption. Compared with trans-syndesmotic fixation, deltoid ligament repair holds the advantage of not needing surgical removal of inferior tibiofibular screws postoperatively.

## Background

Ankle fracture, of all types, is one of the most common injuries in emergency department (1). Lateral malleolus is the most commonly affected area, usually combined with medial and/or posterior malleolus fracture (2, 3). In some specific situations, instead of medial malleolus fragments, deltoid ligament and inferior tibiofibular syndesmotic disruption are found, which is defined as bi- or trimalleolar equivalent ankle fracture or lateral malleolus fracture combined with both deltoid ligament and inferior tibiofibular syndesmotic disruption (4–6). This pattern of ankle fracture leads to medial clear space widening and inferior tibiofibular separation, which belongs to unstable fracture and needs operative intervention.

Mechanically, this pattern of ankle fracture results in ankle instability and decreasing of tibiotalar contact area; therefore, the main purpose of management is to recover ankle stability and tibiotalar contact area (4–6). For operative treatment of lateral malleolus fracture, open reduction and internal fixation (ORIF) is usually suggested. Syndesmotic fixation was first used to stabilize inferior tibiofibular syndesmosis and recover the tibiotalar contact area (7); however, the high occurrence rate of malreduction of the tibiofibular syndesmosis and the need for an extra procedure for removing syndesmotic screws were observed by surgeons (7–10). Therefore, deltoid ligament repair was carried out as a more anatomic method to recover the tibiotalar contact area (11). However, because of the relatively rare type of fracture pattern, there is no consistent conclusion regarding which method is better for the management of deltoid ligament injury and inferior tibiofibular syndesmotic disruption. This study reviewed the patients who received syndesmotic fixation (control group) and deltoid ligament repair (experimental group) and compared the clinical outcomes of these two methods, which could provide evidence for the selection of operative method for the specific type of ankle fracture.

## Methods

### Study design

**Figure 1:**
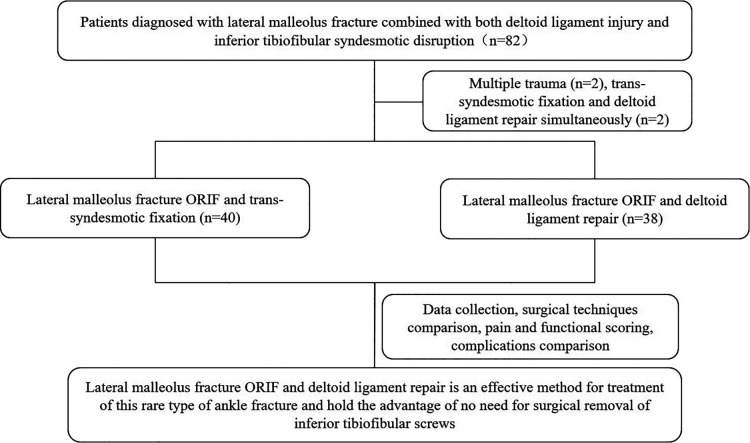
Flow chart for the study cohort.

This is a retrospective study to review the clinical outcomes of patients with lateral malleolus fracture combined with both deltoid ligament and inferior tibiofibular syndesmotic disruption. From 2012 to 2020, patients identified to have lateral malleolus fracture combined with increased medial clear space widening (more than 5 mm with external rotation stress test) or deep deltoid ligament injury in magnetic resonance imaging (MRI) scan and inferior tibiofibular syndesmotic disruption were included. Exclusion criteria were (1) patients with a history of ankle surgery; (2) patients with ankle tumors and/or deformities; (3) patients with chronic ankle instability before injury; (4) patients received other treatment, such as conservative treatment or external fixation, etc.; (5) patients with other severe disease, such as severe osteoporosis, malignant tumor, etc.; and (6) patients with severe soft tissue injury. In total, 82 patients were admitted to the study and 78 (male 44, female 34) patients were included for final analysis (Figure 1); 40 patients who received lateral malleolus ORIF and syndesmotic fixation were included in syndesmotic fixation group (control group), and 38 patients who received lateral malleolus ORIF and deltoid ligament repair were included in deltoid ligament repair (experimental group).

### Surgical techniques

One consistent group of surgeons completed all surgeries. After anesthesia, external rotation stress test was taken under C-arm (GE, United States), medial malleolus clear space wider than 5 mm was confirmed as deltoid ligament injury. Then, fibular fracture was fixed with plate and screws with lateral or posterolateral incision. In the control group, if the medial clear space was still wider than 5 mm with external rotation stress test and inferior tibiofibular syndesmosis drawer test was positive, syndesmotic fixation was carried out. As for the experimental group, if the medial clear space was still wider than 5 mm with external rotation stress test, a medial incision was taken to expose medial capsule and deltoid ligament; after confirmation of the deep deltoid ligament injury, an anchor was placed at the astragalus insertion of deltoid ligament. Simultaneously, two 2-mm holes were drilled at the tibial insertion of deltoid ligament. Then, the ankle was reduced and fixed with a point reduction camp. After confirmation of the reduction of medial ankle and inferior tibiofibular syndesmosis under x-ray, the anchor sutures were traversed the medial malleolus holes and secured at the insertion of deltoid ligament. Superficial layer of deltoid ligament and periosteum were also sutured.

### Perioperative management

Ankle brace or plaster was used postoperatively to keep the ankle in neutral position. Nonsteroidal anti-inflammatory drugs were used for controlling pain. Six weeks after operation, partial weight bearing was allowed with the protection of ankle brace or plaster. Passive movement of ankle and step by step rehabilitation exercises were also allowed with the guidance of a surgeon 6 weeks after operation. In addition, for the control group, inferior tibiofibular screws were moved 6–8 weeks after the primary operation.

### Data collection

The following data were recorded according to hospital case materials: gender, age, height, weight, diagnosis, operation side, operation time, hospital stay, and preoperative comorbidities. Complications (fractures, nerve damage during the operation, deep venous thrombosis, etc.) were recoded according to the case records, outpatient department, or phone follow-up. Visual analog pain scale (VAS) was used to evaluate pain control postoperatively. The Olerud–Molander score and AOFAS Ankle-Hindfoot Scale were used for evaluating functional recovery postoperatively. VAS scores and functional scores were collected according to the outpatient case materials or phone follow-up. All patients were informed about the study. Radiological materials were reviewed and determined in the hospital radiological database.

### Statistical analysis

Quantitative data were recorded as mean ± standard deviation (SD). GraphPad Prism 7.0 (GraphPad software Inc., San Diego, CA, United States) was utilized for statistical analysis. Independent samples *t* tests were used for comparing the experimental and control group scoring at different time points. An analysis of variance (ANOVA) was used to analyze continuous variables, followed by the Tukey–Kramer test. All statistical analyses were two-tailed and a *p* value <0.05 was regarded as statistically significant.

## Results

Forty patients were included in the control group and 38 patients were included in the experimental group. At the final clinic follow-up, no complications or signs of ankle arthritis were recorded in both groups. The average age was 41.53 ± 15.71 years versus 41.13 ± 15.17 years in the experimental group and the control group respectively. Average body mass index (BMI) was 24.54 ± 3.34 kg/m^2^ versus 24.72 ± 3.48 kg/m^2^ in the control group and the experimental group respectively. As shown in Table 1, no statistic difference was found in average age and BMI between two groups. According to the Weber classification, 16 patients in the control group and 11 patients in the experimental group were classified to type B fracture; at the same time, 24 patients in the control group and 27 patients in the experimental group were classified to type C fracture. The injury mechanisms of each group are listed in Table 1; the main injury mechanism of control group was supination-external/eversion rotation (SER, *n* = 20), and the main injury mechanism of experimental group was pronation-external rotation (PER, *n* = 18).

**Table 1:** Demographic characters and diagnosis of trans-syndesmotic screw fixation (control) group and deltoid ligament repair (experimental) group.

	Control group		Experimental group		*p*
	Mean ± SD	*n*	Mean ± SD	*n*	
Age (year)	41.53 ± 15.71	40	41.13 ± 15.17	38	0.9924
BMI (kg/m^2^)	24.54 ± 3.34	40	24.72 ± 3.48	38	0.9924
Gender					* *
Male	—	21	—	23	—
Female	—	19	—	15	—
Weber classification					* *
Type A	—	0	—	0	—
Type B	—	16	—	11	—
Type C	—	24	—	27	—
Injury mechanism					* *
SER		20		8	—
PER		12		18	—
PA		7		10	—
Not known		1		2	—

BMI, body mass index; SER, supination-external/eversion rotation; PER, pronation-external rotation; PA, pronation-abduction.

As for hospital stays, including preoperative hospital stays, postoperative hospital stays, and whole hospital stays, no statistic difference was found between the control and experimental groups. Meanwhile, there was no statistic difference in operation time and intraoperative blood loss between two groups (Table 2).

**Table 2:** Operation time, intraoperative blood loss, and length of hospital stays of trans-syndesmotic screw fixation (control) group and deltoid ligament repair (experimental) group.

	Control group	Experimental group	*p*
	Mean ± SD	Mean ± SD	
Preoperative hospital stays (day)	8.80 ± 2.691	8.54 ± 3.34	*0*.*9924*
Postoperative hospital stays (day)	6.30 ± 3.79	4.90 ± 3.97	*0*.*5202*
Whole hospital stays (day)	15.77 ± 5.64	13.07 ± 3.64	*0*.*09825*
Operation time (min)	105.2 ± 40.10	108.38 ± 50.25	*0*.*9924*
Intraoperative blood loss (ml)	46.50 ± 38.64	37.23 ± 26.58	*0*.*7170*

**Figure 2:**
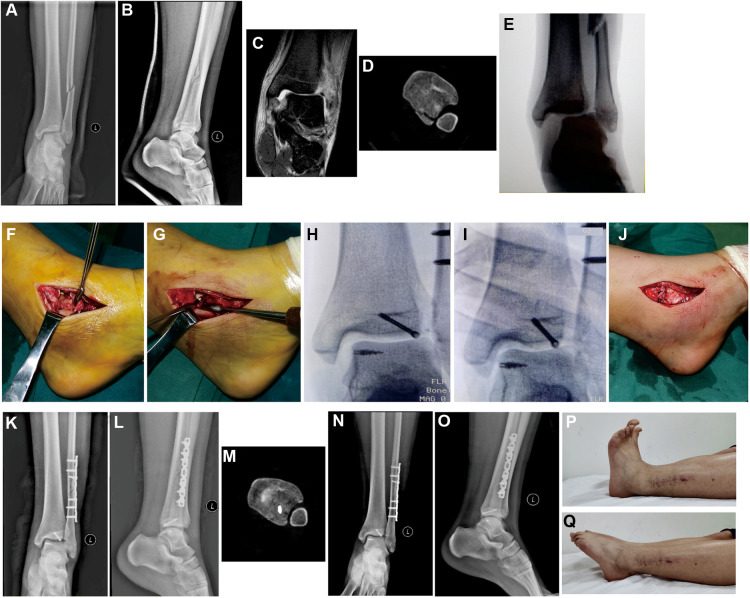
A Male patient diagnosed with Weber type C, Lauge–Hansen PER-IV, trimalleolar equivalent fracture treated with fibula ORIF and suture anchor deltoid ligament repair. Preoperative anteroposterior (**A**) and lateral view (**B**) showed that fibular fracture was above the tibiofibular syndesmosis, and the increased medial malleolus clear space was not obvious with plaster fixation. (**C**) MRI image (without plaster) indicated the deep deltoid ligament injury and increased medial malleolus clear space. (**D**) A CT scan image showed the posterior malleolus fracture and inferior tibiofibular syndesmotic disruption. (**E**) X-ray showed the increased medial malleolus clear space and inferior tibiofibular syndesmotic disruption with external rotation stress test. (**F**) Medial incision was used to show the injured superficial and deep deltoid ligament. (**G**) Suture anchor was placed on the astragalus insertion of deltoid ligament. (**H**) X-ray confirmed the position of suture anchor. (**I**) X-ray confirmed the reduction of medial malleolus clear space and inferior tibiofibular syndesmotic disruption after sutures were tightened. (**J**) Sutured superficial deltoid ligament and periosteum. Postoperative anteroposterior (**K**) and lateral view (**L**) of ankle. (**M)** Postoperative CT scan image to show the reduction of inferior tibiofibular syndesmotic disruption. One year follow-up x-ray examination, anteroposterior (**N**) and lateral view (**O**) showed the union of fracture without displacement. Ankle dorsal extension (**P**) and toe flexion (**Q**) were comparable with uninjured side at 1 year follow-up. PER , pronation-external rotation; ORIF, open reduction and internal fixation; MRI, magnetic resonance imaging; CT, computed tomography.

Figure 2 exhibited a patient with trimalleolar equivalent fracture, which was classified into Weber type C fracture and Lauge–Hansen PER-IV. Preoperative anteroposterior (Figure 2A) and lateral view (Figure 2B) showed fibular fracture and increased medial malleolus clear space. MRI image (Figure 2C) indicated the deep deltoid ligament injury and increased medial malleolus clear space. A computed tomography (CT) scan image (Figure 2D) showed posterior malleolus fracture and inferior tibiofibular syndesmotic disruption. After anesthesia, the increased medial malleolus clear space and inferior tibiofibular syndesmotic disruption became more obvious with external rotation stress test (Figure 2E). After fibular fracture was fixed, a medial incision was taken to show the injured deltoid ligament (Figures 2F,G), a metal suture anchor was placed on the astragalus insertion of deltoid ligament (Figure 2G). X-ray confirmed that the suture anchor was posited in the astragalus insertion of deltoid ligament (Figure 2H), and both medial malleolus clear space and inferior tibiofibular syndesmotic disruption were recovered combined with the recovery of the tibiotalar contact area after suture anchor was knotted (Figure 2I). The superficial layer of deltoid ligament and periosteum were also sutured (Figure 2J). Postoperative anteroposterior (Figure 2K), lateral view (Figure 2L), and CT scan (Figure 2M) showed the reduction of fibular fracture, medial malleolus clear space, and inferior tibiofibular syndesmotic disruption. One year postoperatively, anteroposterior (Figure 2N) and lateral view (Figure 2O) showed the union of fracture without any ankle displacement; ankle dorsal extension (Figure 2P) and toe flexion (Figure 2Q) were comparable with uninjured side.

**Figure 3:**
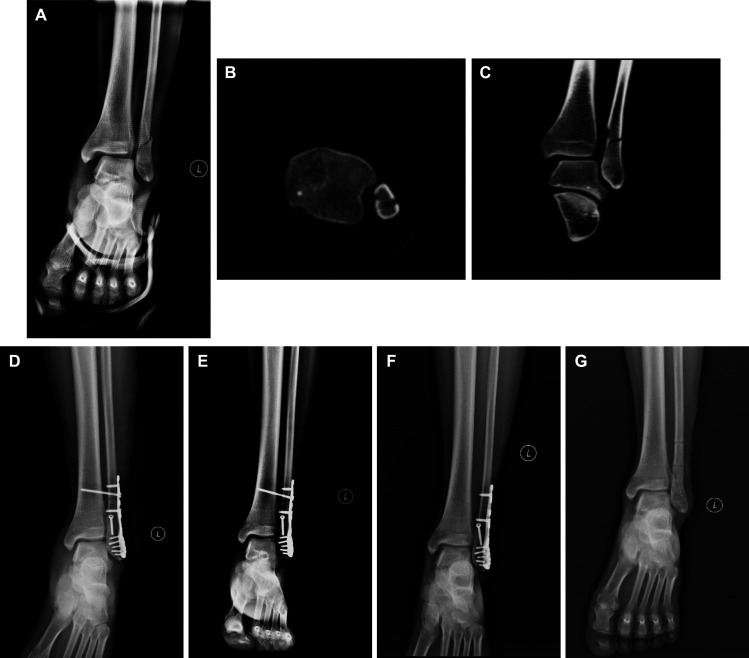
A Male patient with Weber type B, Lauge–Hansen SER-III, bimalleolar equivalent fracture treated with fibula ORIF and trans-syndesmotic screw fixation. Preoperative anteroposterior x-ray image (**A**), axial CT scan image (**B**), and coronal CT (**C**) scan image showed the fibular fracture, increased medial malleolus clear space and inferior tibiofibular syndesmotic disruption. (**D**) Postoperative anteroposterior image showed the reduction of fibular fracture, medial malleolus clear space, and distal tibiofibular joint. One month (**E**), one year (**F**), and post internal fixation removal (**G**) anteroposterior x-ray images did not show any displacement or dislocation, no ankle osteoarthritis was found either. ORIF, open reduction and internal fixation; CT, computed tomography.

Meanwhile, a patient with bimalleolar equivalent fracture, which was classified into Weber type B fracture and Lauge–Hansen SER-III was showed in Figure 3. Preoperative anteroposterior x-ray image (Figure 3A), axial CT image (Figure 3B), and coronal CT image showed that the fibular fracture increased the medial malleolus clear space, posterior malleolus fracture, and inferior tibiofibular syndesmotic disruption. Fibula ORIF was carried out and inferior tibiofibular syndesmotic disruption was fixed with screws. Postoperative anteroposterior x-ray (Figure 3D) showed the reduction of fibular fracture, reduction of medial malleolus clear space, and distal tibiofibular joint. One month (Figure 3E), one year (Figure 3F), and post internal fixation removal (Figure 3G) anteroposterior x-ray images did not show any displacement or dislocation; no ankle osteoarthritis was found either.

**Figure 4:**
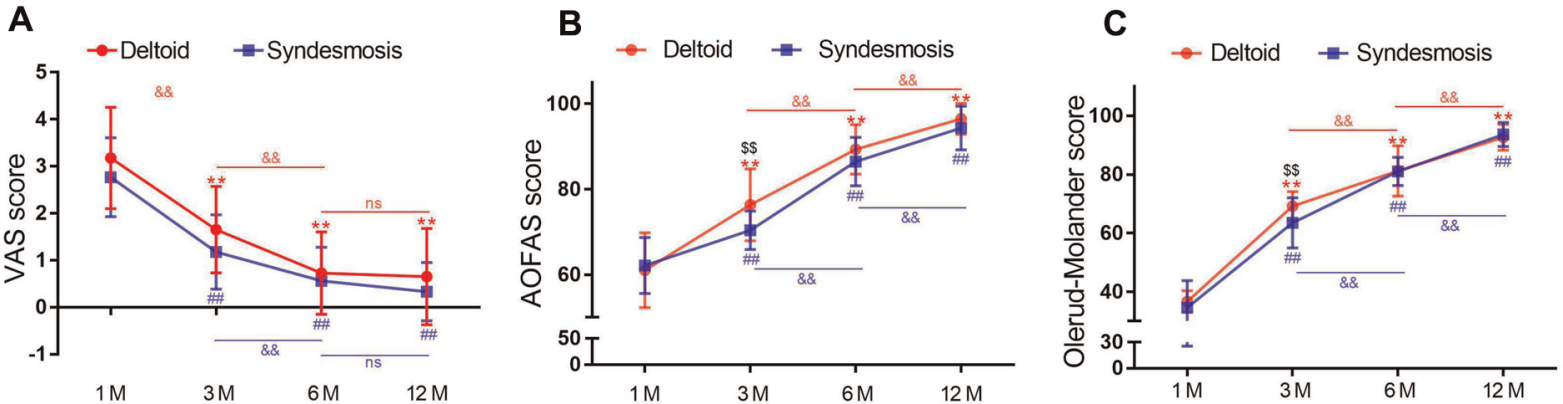
VAS (**A**), AOFAS Ankle-Hindfoot Scale (**B**), and Olerud–Molander scale (**C**) analysis from 1 to 12 months postoperatively. ANOVA: ** *p* < 0.01 compared with 1 month group in experimental group; ## *p* < 0.01 compared with 1 month group in control group; and // *p* < 0.01 compared with the indicated group. Independent sample's *t* tests: // *p* < 0.01 compared with control group. VAS, visual analog pain scale; AOFAS, American Orthopaedic Foot and Ankle Society; ANOVA, analysis of variance;

In addition, according to VAS scoring analysis, from 1 to 12 months postoperatively, both groups obtained obvious decrease of VAS scores, and no statistical difference was found when compare each time point. As for the AOFAS Ankle-Hindfoot scale and Olerud–Molander scale, scores increased gradually from 1 to 12 months postoperatively; meanwhile, scores in the experimental group at three months postoperatively was statistically higher than the control group, although no statistical difference was found in other time points (Figure 4). This might be associated with surgical removal of syndesmotic screws 6–8 weeks postoperatively.

## Discussion

The ankle joint is a rather complex joint which contains a tibial astragalus joint and a syndesmotic joint. Medial collateral ligament (deltoid ligament) and lateral collateral ligament are the main connections of distal tibia and distal fibula to the talus; anterior and posterior lower tibiofibular ligaments are the connections between distal tibia and fibula. Ligaments around the ankle are main stable structures of the ankle. It is well recognized that bimalleolar fractures or trimalleolar fractures are unstable fractures that need ORIF (3, 12). However, in some special situations, instead of bony fracture in medial malleolus, the instability is caused by deltoid ligament injury (13). In the present study, we retrospectively reviewed this rare type of ankle fracture and compared clinical outcomes of deltoid ligament repair versus syndesmotic fixation in lateral malleolus fracture combined with both deltoid ligament injury and inferior tibiofibular syndesmotic disruption.

Some early works suggested that lateral malleolar stability was the key to the whole ankle stability; therefore, fibular ORIF and screw syndesmotic fixation were utilized for the treatment of unstable ankle fracture-associated syndesmotic injury without deltoid ligament repair (7, 14, 15). However, malreduction of the tibiofibular syndesmosis, incongruity of the fibula within the incisura, fibular rotation, etc., were observed on postoperative CT scan, which may contribute to poor functional outcomes (8, 15–17). In addition, screw syndesmotic fixation alters physical biomechanical movement, which needs additional surgery to remove fixation screws. Therefore, more anatomic methods such as tightrope fixation, suture button fixation, etc., for the syndesmotic fixation were carried out and obtained better clinical outcomes compared with screw syndesmotic fixation (9, 10, 18–20). In the other side, recently, the importance of deltoid ligament for the stability of ankle has been identified, and it is believed that repairing injured deltoid ligament at the time of fracture stabilization will obtain better ankle stabilization (11, 21, 22). Furthermore, Massri-Pugin et al. (23) clarified that the deltoid ligament plays an essential role in the stability of syndesmosis. Therefore, according to the “Neer Ring” concept, the ankle joint would be more anatomic stabilization when repair fibular and deltoid ligament ruptures theoretically. In the past few years, for those patients diagnosed with lateral malleolus fracture combined with both deltoid ligament injury and inferior tibiofibular syndesmotic disruption, instead of syndesmotic fixation, we repaired deltoid ligament with suture anchor; we also found that deltoid ligament repair contributes equally for the stabilization of tibiofibular syndesmosis compared with syndesmotic fixation, and no chronic medial malleolus unstable was found with clinical follow-up.

Tibiotalar contact area is an important indicator of ankle reduction (5); anatomical reduction will obtain more tibiotalar contact area and reduce the danger of osteoarthritis. However, it is reported that around 39% patients who received syndesmotic fixation were malreduced (rotational or translational asymmetry) when compared with the uninjured side in CT scan images (8, 16, 17). This may be caused by the lack of an anatomical maker for the reduction of syndesmotic joint. As for the deltoid ligament repair patients, both astragalus and tibia insertions of the deltoid ligament were marked, and the deltoid ligament was repaired anatomically. Reduction of inferior tibiofibular syndesmosis and medial malleolus clear space were also confirmed intraoperatively. We observed better reduction of tibiofibular syndesmosis in deltoid repair patients compared with syndesmotic fixation patients through postoperative CT scan images.

Our study found that both deltoid ligament repair versus syndesmotic fixation obtained good clinical outcome 6 and 12 months after operation. No complications or osteoarthritis was found with long-term follow-up. All patients removed syndesmotic fixation screws 6–8 weeks after operation, as research suggested that removal of syndesmotic fixation screws would benefit in restoring the malreduction of syndesmosis and finally achieve anatomic reduction of the distal tibiofibular joint (17, 24). According to our pain scoring and functional scorings, it seems that the deltoid repair group obtained better clinical results at the early stage postoperatively. Jones et al. (25) retrospectively compared the clinical outcomes of deltoid ligament repair versus syndesmotic fixation in bimalleolar equivalent ankle fractures and found no obvious difference between two groups, which is similar to our results. However, there are several differences between this study and our study. First, both Weber type C and type B fractures were included in our study, and most type C fracture patients were in the experimental group with PER injury mechanism, which was complementary for the indications of deltoid ligament repair. Second, the position site of suture anchor was different, and we placed the anchor at the astragalus insertion of deltoid ligament and drilled two holes at tibial insertion of deltoid ligament, which applied stronger primary stability. Third, our study applied some evidence for deltoid ligament repair for the treatment of trimalleolar equivalent fractures.

Posterior malleolar fractures are of prognostic relevance in ankle fracture dislocations; the size of the posterior malleolus fracture fragment and the size of the posterior malleolus affect the outcome of posterior malleolar fracture (26, 27). However, it is still controversial in the management of posterior malleolus fractures. In our study, posterior fracture fragment was fixed with closed screw if the reduction is acceptable and fracture fragment is big enough for fixation.

Our study has some limitations. First, the study was limited by the small simple size, with the recognition of the possibility of deltoid ligament injury rather than medial malleolus fragment, and a larger multicentered randomized retrospective study needs to be carried out. However, pilot studies are also necessary for applying some evidence to further utilize a new method. Second, because of the rarity of this pattern of ankle fracture, cases are distributed from 2012 to 2020, which may affect the uniformity of the clinical outcomes. Third, this is a retrospective study which is limited by the nature of this type of study.

## Conclusions

Deltoid ligament repair is an effective method for the treatment of lateral malleolus fracture combined with both deltoid ligament injury and inferior tibiofibular syndesmotic disruption, which could recover the anatomical alignment of ankle joint, avoid extra operation for syndesmotic screws removal, and obtain better early clinical outcome compared with syndesmotic fixation.

## Data Availability

The raw data supporting the conclusions of this article will be made available by the authors, without undue reservation.
